# Saccadic Adaptation Is Associated with Starting Eye Position

**DOI:** 10.3389/fnhum.2016.00322

**Published:** 2016-06-27

**Authors:** Svenja Gremmler, Markus Lappe

**Affiliations:** ^1^Department of Psychology, University of MünsterMünster, Germany; ^2^Otto-Creutzfeldt Center for Cognitive and Behavioural Neuroscience, University of MünsterMünster, Germany

**Keywords:** saccadic adaptation, eye position signal, motor learning, oculomotor control, gain fields

## Abstract

Saccadic adaptation is the motor learning process that keeps saccade amplitudes on target. This process is eye position specific: amplitude adaptation that is induced for a saccade at one particular location in the visual field transfers incompletely to saccades at other locations. In our current study, we investigated wether this eye position signal corresponds to the initial or to the final eye position of the saccade. Each case would have different implications on the mechanisms of adaptation. The initial eye position is not directly available, when the adaptation driving post saccadic error signal is received. On the other hand the final eye position signal is not available, when the motor command for the saccade is calculated. In six human subjects we adapted a saccade of 15 degree amplitude that started at a constant position. We then measured the transfer of adaptation to test saccades of 10 and 20 degree amplitude. In each case we compared test saccades that matched the start position of the adapted saccade to those that matched the target of the adapted saccade. We found significantly more transfer of adaptation to test saccades with the same start position than to test saccades with the same target position. The results indicate that saccadic adaptation is specific to the initial eye position. This is consistent with a previously proposed effect of gain field modulated input from areas like the frontal eye field, the lateral intraparietal area and the superior colliculus into the cerebellar adaptation circuitry.

## 1. Introduction

Saccades are fast eye movements which shift the retinal area of highest receptor density, the fovea, from one point of interest to another one. These movements are so fast that visual feedback can not be fully processed while the gaze is in flight. Therefore, the motor signal that steers the movements has to be prepared well in advance. Due to alterations in the oculomotor plant by growing or aging or due to a changed response behavior of the plant by muscle fatigue a fixed motor command would lead to dysmetric saccades after some time. For this reason, the motor signal steering the movement is continuously adjusted. This motor learning can be induced in the laboratory employing the McLaughlin paradigm (McLaughlin, 1967). Using eye tracking devices the target is shifted while the saccade is in mid flight. The error signal that drives the adaptation is the post-saccadic error signal, the distance between the detected target position on the retina and the expected target position (Wong and Shelhamer, 2011; Collins and Wallman, 2012; see Herman et al., 2013 for a review).

Several studies have investigated if the amplitude modifications that are induced at one spatial location, are transferred completely to other locations, i.e., if saccadic adaptation is eye position specific. Early studies saw no influence of the eye position on the adaptation state of a saccade (Semmlow et al., 1989; Frens and Van Opstal, 1994; Albano, 1996), suggesting a retinal reference frame of saccadic adaptation. Later studies have shown that saccades of the same direction and amplitude can be adaptively shortened and lengthened simultaneously if the spatial location of the saccade is changed (Shelhamer and Clendaniel, 2002; Alahyane et al., 2004). This finding in the so called differential adaptation paradigm led to the conclusion that saccadic adaptation cannot be encoded in a pure retinal reference frame. Furthermore, recent studies have revealed that saccadic adaptation is eye position specific (Tian and Zee, 2010; Havermann et al., 2011; Zimmermann and Lappe, 2011; Zimmermann et al., 2011; Wulff et al., 2012) the way that adaptation which is induced at one eye position is not completely transferred to other eye positions. The results presented by Havermann et al. (2011) provide a possible explanation for the full transfer of adaptation to untrained eye positions that was observed in earlier studies (Semmlow et al., 1989; Frens and Van Opstal, 1994; Albano, 1996). Adaptation that was induced with a central eye position was transferred completely to other locations, while the transfer of adaptation that was induced with deflected eye positions is modulated by eye position. Thus, if during the adaptation phase saccades were executed in the central field, little or no modulation of adaptation transfer by eye position is expectable.

The recently revealed eye position specificity in saccadic adaptation indicates that the adaptation mechanism considers and processes the information provided by the eye position signal. This finding opens the question, whether adaptation depends on the eye position before the saccade, the initial eye position, or wether it depends on the eye position after the saccade is finished, the final eye position. By investigating this question, we can support a more comprehensive understanding of the adaptation mechanism because the two possibilities would lead to different prerequisites of the physiological system: If adaptation is assigned to the initial eye position, the eye position signal would not be directly available at the same time as the error signal that drives adaptation which can be calculated only after the saccade is finished. Thus information about the initial eye position would need to be maintained over time for the adaptation adaptation. On the other hand, if the final eye position, which is available simultaneously with the post-saccadic error signal, were to be used, complications arise in the preparation of subsequent saccades to the same target. These saccades should become adapted but information about the final eye position and thus the possible gain modifications is not available when the motor command is prepared.

To test if the initial or the final eye position signal is used in saccadic adaptation, we need to measure the transfer of adaptation from one adapted saccade to saccades matching its initial or final eye position, respectively. If one eye position is altered while the other eye position remains unchanged, the test saccade has to have a different amplitude than the adapted saccade. Since adaptation of any particular saccade amplitude is known to transfer partially to saccades with smaller or larger amplitudes, a characteristic of saccadic adaptation called the adaptation field (Frens and Van Opstal, 1994; Collins et al., 2007; Schnier et al., 2010), varying only one of the eye positions will automatically lead to reduced transfer of adaptation. We accounted for this superimposed effect by comparing the adaptation state of two test saccades with identical amplitude, that was different from the adapted saccade's amplitude. One of the test saccades then had the same start position and the other test saccade had the same target position as the adapted saccade. A further complication is that the final eye position is not identical to the target position (because of saccade hypometry Becker, 1989) and changes during adaptation as saccade amplitude becomes smaller. However, the predictions for adaptation transfer are the same since test saccades with the same target location as the adapted saccade will have final eye positions closer to the final eye position of the adapted saccade compared to the test saccades with different target positions. However, we will analyze the amount of transferred adaptation with regard to start and target position as well as with regard to the actual saccade amplitude. Thus with matching the start or the target position of the test saccades to those of the adapted saccade, we can observe if more adaptation is transferred to saccades sharing the initial eye position or the final eye position, respectively.

## 2. Materials and methods

Six subjects (3 women, 3 men, mean age 21.8 ± 2.1) participated in the experiment. The subjects were seated in a dark room at a distance of 57 cm in front of a 22′′ monitor (Eizo FlexScan F930, resolution of 1280 × 1024 pixels, refresh rate 100 Hz). The monitor screen thus corresponded to a visual field of 40 × 30 degree. The stimuli that were presented on the screen were filled white circles with a diameter of 0.25 degree and a luminance of 0.5 cd/m^2^.

For eye movement recording and analysis we used the EyeLink1000 system (SR research). The right eye was recorded in every subject with 1k Hz sampling rate. Start and end of saccades were tagged when eye velocity exceeded or went below a threshold of 30 deg/s and acceleration exceeded or fell below a threshold of 8000 deg/s^2^. For stimulus presentation and data analysis we used MATLAB with the psychtoolbox extension (Brainard, 1997). The experiment was performed in accordance with the principals and ethical standards laid down in the 1964 Declaration of Helsinki and approved by the local ethics committee.

### 2.1. Behavioral task

**Figure 1:**
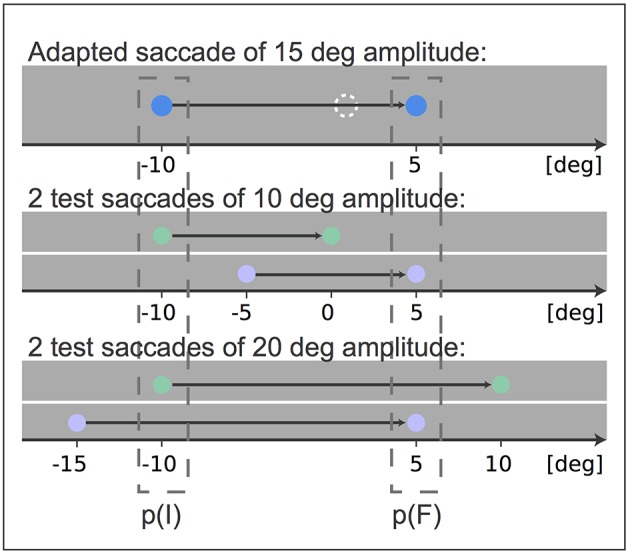
**Experimental setup: After adaptation of a 15 degree amplitude saccade the transfer of adaptation to two saccades of 10 degree amplitude and to two saccades of 20 degree amplitude was tested**. The blue points indicate the fixation point and target stimulus of the adapted saccade and the dashed white circle indicates the shifted target position that induces adaptation. The test saccades matched either the fixation position p(I) or the target position p(F) of the adapted 15 degree amplitude saccade. The fixation point and target stimulus of test saccades matching p(I) are indicated in green and the fixation point and target stimulus of test saccades matching p(F) are indicated in purple. All stimuli were white during the experiment. The black arrow indicates the gaze movement.

We adapted a saccade of 15 degree amplitude with an intra-saccadic target step of 4 and 6 degree against the saccade direction, respectively. Afterwards the adaptation state of two 10 degree amplitude saccades were tested. While one saccade had the same initial eye position, i.e., fixation position, as the adapted saccade, the other saccade had the same target position and hence similar final eye positions. In the same way we tested the adaptation states of two 20 degree amplitude saccades, one saccade with the same start position as the 15 degree adapted saccade and the other with the same target position (Figure 1). The fixation position of the adaptation saccade of 15 degree amplitude was at −10 degree horizontal gaze angle and on eye level. The first of the 10 degree saccades started at the same position, the other one started from −5 degree. Analogously, the first of the 20 degree saccades started at −10 degree horizontal gaze angle and the other started at −15 degree horizontal gaze angle. Thus, we had five different types of saccades, one adaptation saccade, two saccades with the same start and thus initial eye position p(I) as the adapted saccade and two saccades with the same target position and thus final eye position p(F) as the adapted saccade.

The session started with a block of 100 pre-adaptation trials with 20 saccades of each type in a randomized order. In pre-adaptation trials a fixation point was presented. The subjects were instructed to saccade to this point and keep it fixated. After a random time between 0.8 and 2.5 s, in which the subjects fixation was controlled, the fixation point disappeared and a target was presented. The subjects was requested to make a saccade to the target as soon as the target appears. After the saccade, the target remained visible for 1.5 s. The second block consisted of 150 adaptation trials. In an adaptation trial the target was shifted against the direction of the saccade when the saccade was in mid-flight. The target shift occurred after the gaze had traveled 3 degree in direction of the target. In all adaptation trials the amplitude was 15 degree and the saccade started at −10 degree horizontal gaze angle. The target stepped back 4 degree in the first 75 adaptation trials and 6 degree in the second 75 adaptation trials. The adaptation block also contained 50 randomly interspersed trials in which the target was presented 10 degree above the fixation point, rather than 15 degree to the right, in order to prevent the subjects from preplanning and stereotyping the saccade. The target did not shift in these vertical trials. The last block of the session, the test block, consisted of 200 trials. 100 trials were conventional adaptation trials to prevent adaptation loss. The other 100 consisted of 20 test trials of each saccade type. In these test trials the target was shown at the respective target position and was switched off when the saccade onset was detected to avoid feedback about the performance to the saccadic system.

## 3. Results

We tested the eye position specificity of saccadic adaptation in two conditions. For both conditions we initially adapted a 15 degree saccade (the trained saccade). Afterwards we compared the adaptation states of two 20 degree test saccades with different start and target positions (first condition) and of two 10 degree test saccades with different start and target positions (second condition). The two spatial positions of the saccades were chosen in this way to test saccades that either had the same initial eye position as the adapted 15 degree saccade or a comparable final eye position (Figure 1). If the adaptation of the 15 degree trained saccade is assigned to its initial position, then the saccades with the same start positions (Figure 1: green saccades) should show stronger adaptation than the test saccades with different start positions (Figure 1: purple saccades). On the other hand, if the adaptation of the 15 degree trained saccade is assigned to its final eye position, then the saccades with the same target positions (Figure 1: purple saccades) should show stronger adaptation, since the final eye positions of these saccades are much closer to the final eye position of the trained saccade before the adaptation. Even if we consider the final eye positions after the adaptation, at least the purple 20 degree test saccade has an end position closer to that of the trained blue saccade. Thus, if adaptation is assigned to the final eye position, stronger adaptation of the purple test saccade was to be expected.

We used the pre-adaptation trials and the post-adaptation test trials of each saccade type to calculate the mean baseline amplitude *A*_*pre*_ and the mean post-adaptation amplitude *A*_*post*_, respectively. We excluded trials from the analysis in which the saccade was started in the time interval of 0–90 ms after target presentation and we excluded trials in which the executed saccade had an amplitude of less than 3.5 degree. That occurred in less than 1 % of the trials. The amplitude change AC of the adapted 15 degree saccade and the amount of adaptation that was transferred to the four test saccades, two matching the initial eye position p(I) of the 15 degree saccade and the other two matching the final position, p(F), was calculated as following

$$\begin{array}{lll} {\text{AC}}_{15} & = & A_{pre,15}-A_{post,15}\\ {\text{AC}}_{10,p\left(I\right)} & = & A_{pre,10,p\left(I\right)}-A_{post,10,p\left(I\right)}\\ {\text{AC}}_{10,p\left(F\right)} & = & A_{pre,10,p\left(F\right)}-A_{post,10,p\left(F\right)}\\ {\text{AC}}_{20,p\left(I\right)} & = & A_{pre,20,p\left(I\right)}-A_{post,20,p\left(I\right)}\\ {\text{AC}}_{20,p\left(F\right)} & = & A_{pre,20,p\left(F\right)}-A_{post,20,p\left(F\right)} \end{array}$$

Due to the adaptation field, the two 10 degree test saccades should show less adaptation than the two 20 degree test saccades, thus the total offset between the two 10 degree test saccades should also be smaller. Therefore, we calculated the offset normalized to the mean adaptation state of the two 10 degree test saccades and the two 20 degree test saccades, respectively, to see if the effect of eye position is of equal size in both conditions. The normalized offset between the two adaptation states of the same sized test saccades δ*S* was calculated as:

$$\begin{array}{lll} \delta S_{10} & = & \frac{{\text{AC}}_{10,p\left(I\right)}-{\text{AC}}_{10,p\left(F\right)}}{{\text{AC}}_{10,p\left(I\right)}+{\text{AC}}_{10,p\left(F\right)}}\\ \delta S_{20} & = & \frac{{\text{AC}}_{20,p\left(I\right)}-{\text{AC}}_{20,p\left(F\right)}}{{\text{AC}}_{20,p\left(I\right)}+{\text{AC}}_{20,p\left(F\right)}} \end{array}$$

**Figure 2:**
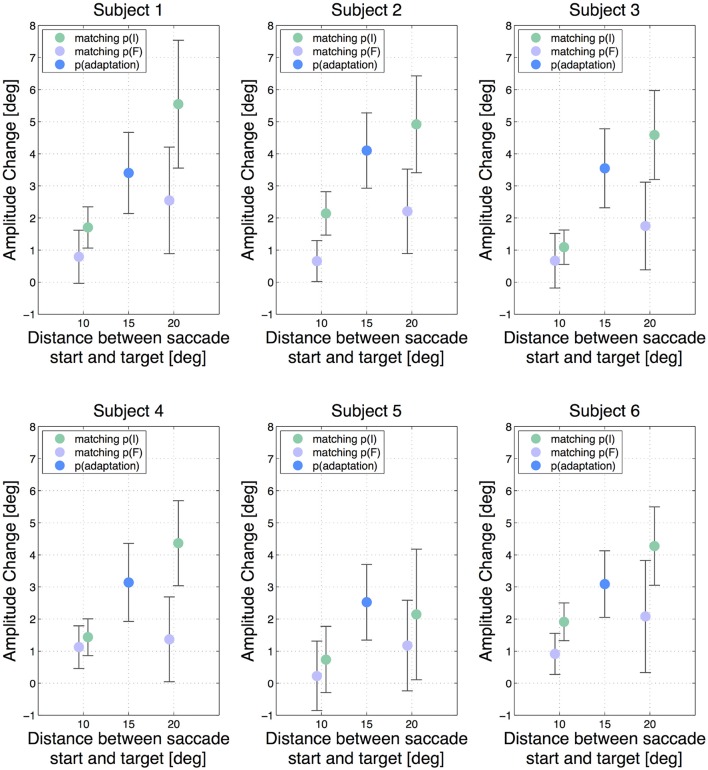
**Amplitude changes of all subjects for all 5 saccade types**. The error bars show the standard deviations. The blue dot indicates the gain change measured in the adapted 15 degree saccade. The green dots show the gain change in the saccades that had the same initial eye position p(I) as the adapted 15 degree saccade and the purple dots show the results for saccades having the same target position p(F). The horizontal offset between the two 10 degree saccades and the two 20 degree saccades, respectively, has been added manually to improve lucidity. A vertical difference between the green and the purple dots in one test saccade type indicates an offset in adaptation transfer.

**Figure 3:**
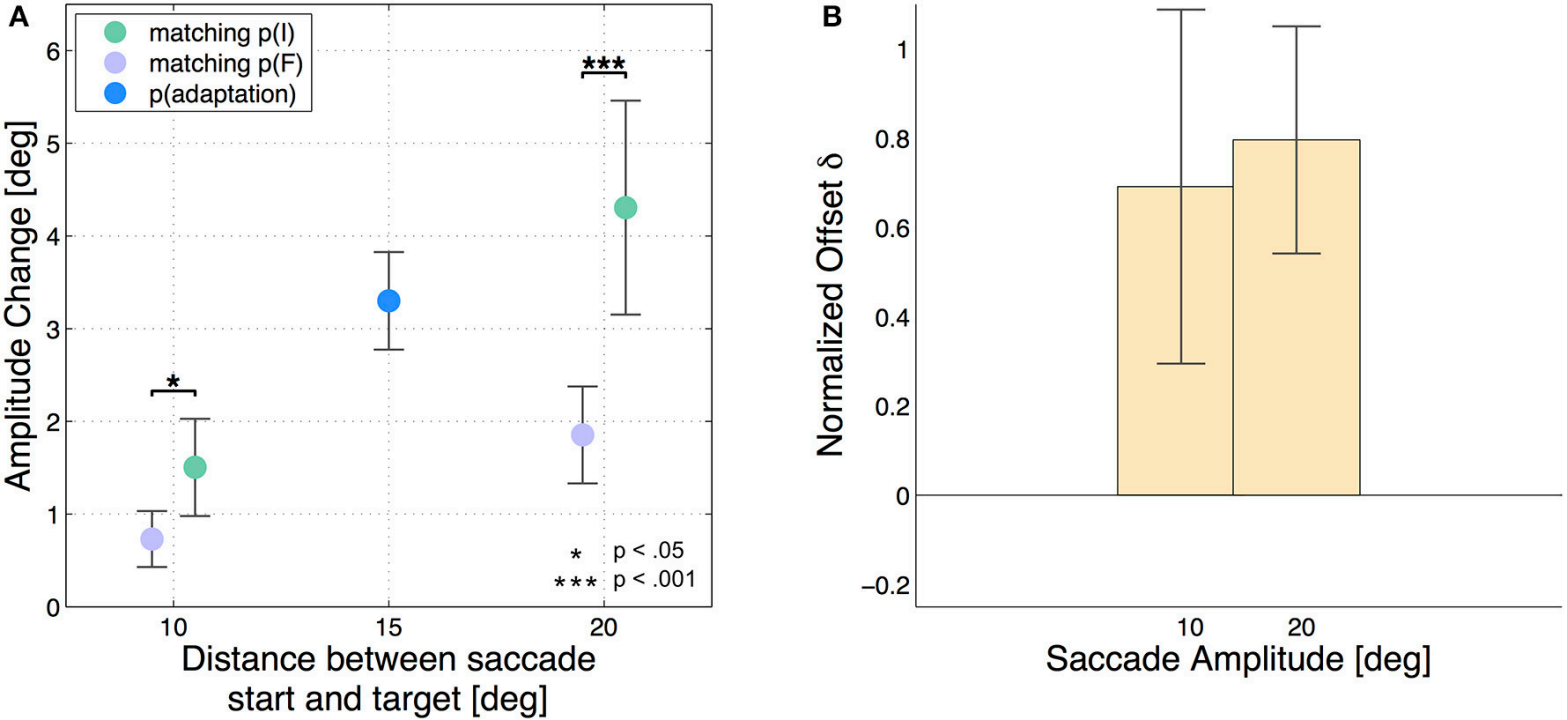
**Mean results of all six subjects**. The error bars show the standard deviations. **(A)** Averages of amplitude change over the six subjects for all 5 saccade types. The blue dot indicates the gain change measured in the adapted 15 degree saccade (AC_15_ = 3.3 ± 0.5 degree). The green dots show the gain change in the saccades that had the same initial eye position p(I) like the adapted 15 degree saccade (AC_10, *p*(*I*)_ = 1.5 ± 0.5 degree and AC_20, *p*(*I*)_ = 4.3 ± 1.2 degree) and the purple dots show the results for saccades matching the final eye position p(F) (AC_10, *p*(*F*)_ = 0.7 ± 0.3 degree and AC_20, *p*(*F*)_ = 1.9 ± 0.5 degree). The test saccades of 10 degree amplitude show considerably less adaptation than the 20 degree test saccades. Furthermore, the saccades with the same saccade target positions p(F) like the adaptation saccade show significantly less adaptation than the saccades which were started at the same fixation position like the adaptation saccade in the case of the 20 degree saccades as well as in the case of the 10 degree saccades. Again the little horizontal offset has been added manually. **(B)** The bars present the normalized offset in adaptation state δS_*type*_ between the test saccades started at p(I) and the test saccades ending at p(F).

Hence, a positive result indicates that a higher amount of adaptation was transferred from the 15 degree saccade to the test saccade having the same initial position p(I) than to the test saccade with the same final position p(F). The analysis was performed independently for the two 10 degree test saccades and for the two 20 degree test saccades. Figure 2 shows the results of each subject with the adaptation states of all 5 saccade types with respect to the start and target position of the saccades, while Figure 3A shows the mean adaptation states of all six subjects for all 5 saccade types. The averaged results show that the two 20 degree saccades are adapted to a comparable degree as the 15 degree adaptation saccade, whereas the two 10 degree saccades are much less adapted. This resembles the adaptation fields described by Frens and Van Opstal (1997) and Collins (2007). Furthermore, for the 10 degree test saccades, those saccades with the same start positions are adapted to a higher extent than the test saccades with the same target positions (two-tailed *t*-test, *p* = 0.01). This means that more adaptation is transferred from the 15 degree saccade to the 10 degree saccade in the case that the start positions of the two saccades are identical, whereas less adaptation is transferred if the target positions of the two saccades are identical. The results of the two 20 degree test saccades show the same effect (two-tailed *t*-test, *p* < 0.001). Hence, we find more transfer of adaptation to test saccades with the same start positions (green trials) than to test saccades with the same target positions (purple trials) in both conditions. Furthermore, the normalized offsets are of comparable size in the two conditions: δ*S*_10_ = 0.7 ± 0.4 and δ*S*_20_ = 0.8 ± 0.3. The mean normalized offset in the adaptation states δS of all subjects is presented in Figure 3B.

**Figure 4:**
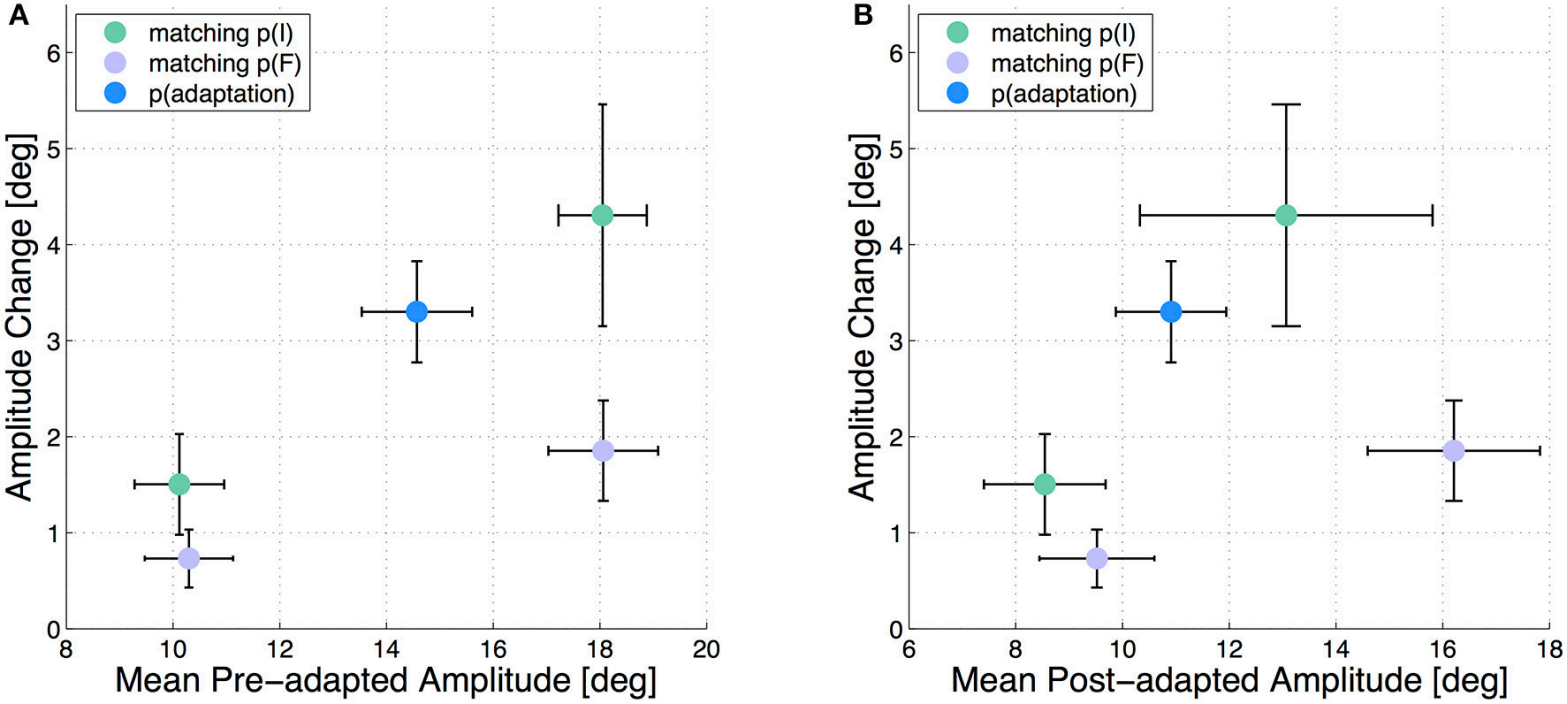
**Mean adaptation states of the 5 test saccades in all six subjects**. The error bars show the standard deviations. The amplitude change in each test saccade is plotted against the mean pre-adapted amplitude of the respective test saccade **(A)** and against the mean post-adapted amplitude **(B)**.

Additionally to the described analysis regarding the start and target positions, we depict the adaptation state of the test saccades with respect to the real pre- and post-adaptation amplitudes. The results are presented in Figure 4 and support the finding, that those test saccades with the same start position are stronger adapted than the test saccades with the same target position.

We wanted to assure that the different adaptation states of saccades with the same amplitude but different starting positions are not caused by systematic differences in saccade execution at the different positions. Thus, we compared the amplitudes of all subjects in the pre-adaptation phase in the 10 degree saccades at one starting position with the 10 degree saccades at the other starting position and we compared the amplitudes of the 20 degree saccades at one starting position with the 20 degree saccades at the other starting position. We neither found a significant difference between the pre-trials with 10 degree amplitude matching the initial eye position of the adaptation saccade and those matching the target position of the adaptation saccade (two-tailed *t*-test, *p* = 0.2) nor a significant difference between the pre-trials with 20 degree amplitude matching the initial eye position of the adaptation saccade and those matching the target position of the adaptation saccade (two-tailed *t*-test, *p* = 0.9). Therefore, the different adaptation states we found after the adaptation phase between saccades matching the start or target position of the 15 degree saccade could not originate from adaptation field effects.

## 4. Discussion

In this study we investigated wether saccadic adaptation is assigned to the eye position from which saccades are started or to the final eye position at the end of these saccades. Therefore, we adapted a saccade of 15 degree amplitude and tested the transfer of adaptation to saccades that either matched the fixation position or the target position of the trained saccade. The results clearly show that a larger amount of adaptation is transferred to the saccades with the same initial eye position as the adapted 15 degree saccade. That means the adaptation is assigned to the saccade start position during adaptation.

In previous studies, we proposed a possible mechanism based on eye position gain fields which may underlie the general eye position specificity in saccadic adaptation (Havermann et al., 2011; Wulff et al., 2012). The configuration of this proposed mechanism might also explain the assignment of the amplitude modification to the initial eye position. The cerebellum is a crucial structure for inward adaptation of reactive saccades (see Pelisson et al., 2010 for a review). Furthermore, in the cerebellum the motor command modifications might be restricted to that neuronal input composition, which was received by the cerebellum during the motor learning (Edelman and Goldberg, 2002). This way a saccade's amplitude is only effectively influenced by prior adaptation if the active input to the cerebellum during the generation of that saccade resembles the active input during the generation of a previously inaccurate, and thus adapted, saccade. On the one hand, the neurons that fire in relation with the generation of a saccade are determined by the saccade amplitude in many brain areas, for example in the superior colliculus, the frontal eye field and the lateral intraparietal area. If now the test saccade has a different amplitude than the adapted saccade, the neuronal input to the cerebellum during saccade generation will also be different. Experiments show that indeed a test saccade with a differing amplitude shows less adaptation than the adapted saccade (Frens and Van Opstal, 1994; Collins et al., 2007; Schnier et al., 2010). On the other hand, the neuronal input composition to the cerebellum is also influenced by gaze direction during saccade generation. This results from the occurrence of so called eye position gain fields, which modulate a neuron's firing rate by the current position of the eye (Andersen and Mountcastle, 1983; Zipser and Andersen, 1988). The neuron then responds to both the retinal target location as well as the current eye position. The eye position modulation has the form that the cell response varies monotonically with the initial eye position in the orbit. Mathematically, the response of a neuron with an eye position gain field modulation can be approximated by the product of a Gaussian function of retinal target position and a sigmoid function of initial eye position (Pouget and Sejnowski, 1997). Neurons with such eye position gain fields occur in several areas of the oculomotor pathway, like in the fastigial nucleus (Fuchs et al., 1993), the nucleus reticularis tegmenti pontis (NRTP) (Crandall and Keller, 1985), the superior colliculus (SC) (Van Opstal et al., 1995; Campos et al., 2006), the lateral intraparietal area (LIP) (Andersen et al., 1990), the frontal eye field (FEF) (Cassanello and Ferrera, 2007), area V3A (Galletti and Battaglini, 1989) and area V6A (Galletti et al., 1995). Hence, the composition of the target command that is received by the cerebellum includes output of areas with gain field modulation like the FEF, LIP, and SC. Thus, if a test saccade has the same start position as the trained adapted saccade, the input to the cerebellum during saccade generation is more similar between test and adaptation saccade as if the initial eye positions differ, due to the gain field modulation. Therefore, the test saccade with the same starting position as the adapted saccade should be influenced to a larger degree by the modification of the motor command during the adaptation. The result we present here is in good accordance with this prediction of the gain field based mechanism of eye position specificity in saccadic adaptation since we found a higher amount of adaptation being transferred to the test saccades with the same starting eye position.

We conclude that saccadic adaptation is specific to the initial eye position of the saccades during adaptation. This behavior resembles the predictions made based on the eye position gain field model in saccadic adaptation previously suggested (Havermann et al., 2011; Wulff et al., 2012).

## Author contributions

Conception and design of the experiment, data analysis: SG. Interpretation of the data, revising the content, final approval of the version: SG, ML.

## Funding

This work was supported by the German Science Foundation DFG LA-962/6.

### Conflict of interest statement

The authors declare that the research was conducted in the absence of any commercial or financial relationships that could be construed as a potential conflict of interest.
